# Computed tomography–based device-sizing in Amplatzer Amulet left atrial appendage occlusion

**DOI:** 10.1007/s10840-023-01665-y

**Published:** 2023-10-26

**Authors:** Jonatan Gerard Nirmalan, Anders Kramer, Kasper Korsholm, Jesper Møller Jensen, Jens Erik Nielsen-Kudsk

**Affiliations:** Department of Cardiology, Aarhus University Hospital, Palle Juul-Jensens Boulevard 99, Aarhus N, 8200 Aarhus, Denmark

**Keywords:** Amplatzer Amulet, Left atrial appendage, Left atrial appendage occlusion, Cardiac computed tomography, Peri-device leak

## Abstract

**Background:**

Amplatzer Amulet is a frequently used device for left atrial appendage occlusion (LAAO). The current sizing protocol is based on the maximum diameter of the left atrial appendage (LAA) landing zone. However, mean, perimeter-, or area-derived diameter might be more accurate measures for device sizing.

**Methods:**

Retrospective analysis of 150 consecutive patients undergoing LAAO is guided by pre-procedural cardiac CT. A total of 117 patients were included; 7 were excluded due to renal failure and 26 due to closure with the sandwich technique. The maximum, mean, area-, and perimeter-derived diameters of the landing zone were derived from pre-procedural cardiac CT scans, and their accuracy to predict the implanted device size was investigated. The predicted device size was determined based on the currently recommended sizing algorithm. Peri-device leak (PDL) was assessed (grade 1–3) along with the underlying mechanism.

**Results:**

Device-sizing accuracy was superior for mean, area-, and perimeter derived diameters compared with the maximal diameter, especially for eccentric landing zones. Mean difference between predicted and actually implanted device size was 0.08 mm (± 2.77), 0.30 mm (± 2.40), − 0.39 mm (± 2.43), and − 2.55 mm (± 2.57) across mean, area-derived, perimeter-derived, and maximal diameter, respectively. Grade 3 peri-device leak was seen in 8.5% of implants without a significant association to the eccentricity of the landing zone. The leading mechanism for PDL was device malalignment.

**Conclusion:**

Our results indicate mean, area-, and perimeter-derived diameters of the device landing zone to perform similar and superior in device-sizing accuracy compared with the maximum diameter.

**Graphical Abstract:**

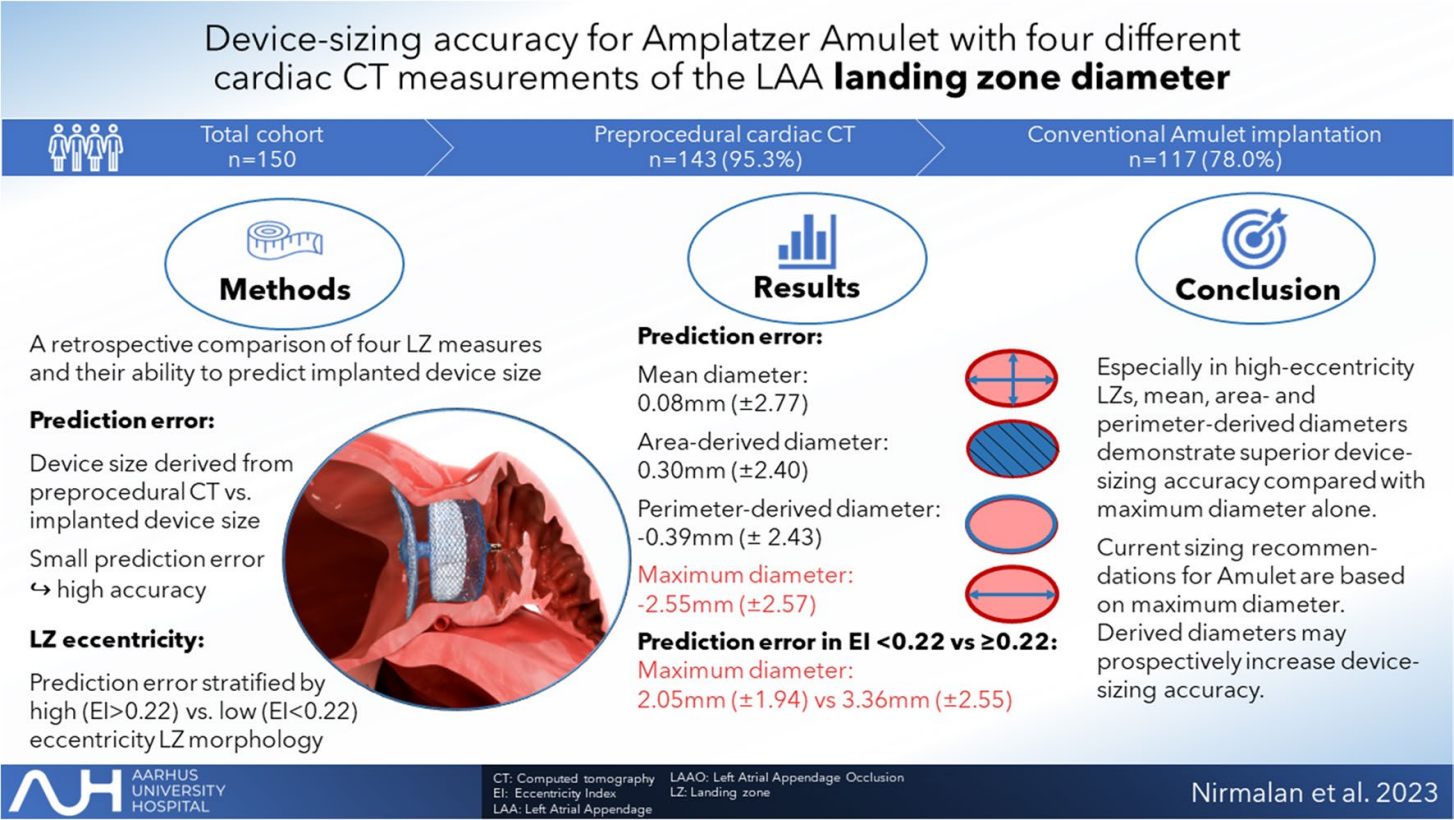

**Supplementary Information:**

The online version contains supplementary material available at 10.1007/s10840-023-01665-y.

## Introduction

Left atrial appendage occlusion (LAAO) has become an alternative stroke prevention strategy in atrial fibrillation (AF) among patients unsuitable for long-term anticoagulation [1–3].

The geometric variability of the LAA is huge and renders device-sizing complex [4–6]. Nevertheless, accurate device sizing is crucial to achieve an optimal LAA closure result. The consequences of inaccurate device sizing may be extra implantation attempts, longer procedure time [7], higher risk of device embolization, and residual peri-device leak (PDL). The latter has become an area of particular focus, as PDL may be associated with an increased risk of thromboembolic events [8–10].

Device sizing has been based on the maximum diameter of the LAA landing zone using transesophageal echocardiography (TEE). However, cardiac computed tomography (CT) is used increasingly for pre-procedural imaging. Data indicate that it might lead to more successful device implantations than a TEE-guided strategy [7, 11, 12]. Moreover, CT can be used for printing of patient-specific 3D-models of the LAA or simulation of implant result [13]. In addition to detailed 3D representations of the LAA morphology, cardiac CT allows for determination of the mean, area-, and perimeter-derived diameters of the LAA device landing zone. Compared to the maximum diameter, these calculated measures may prove more accurate for device sizing, particularly as most landing zones are eccentric [14–16]. In theory, mean, area-derived, and perimeter-derived diameter more accurately represent the geometrical conditions of eccentric landing zones, accounting for both the largest and smallest dimensions of the landing zone (Supplemental Fig 1). In this study, we aimed to investigate different measures of the LAA landing zone and their ability to predict the implanted size of the Amplatzer Amulet device. Secondarily, we attempted to assess the association between LAA eccentricity, sizing, and PDL.

## Materials and methods

### Study design and population

**Fig. 1:**
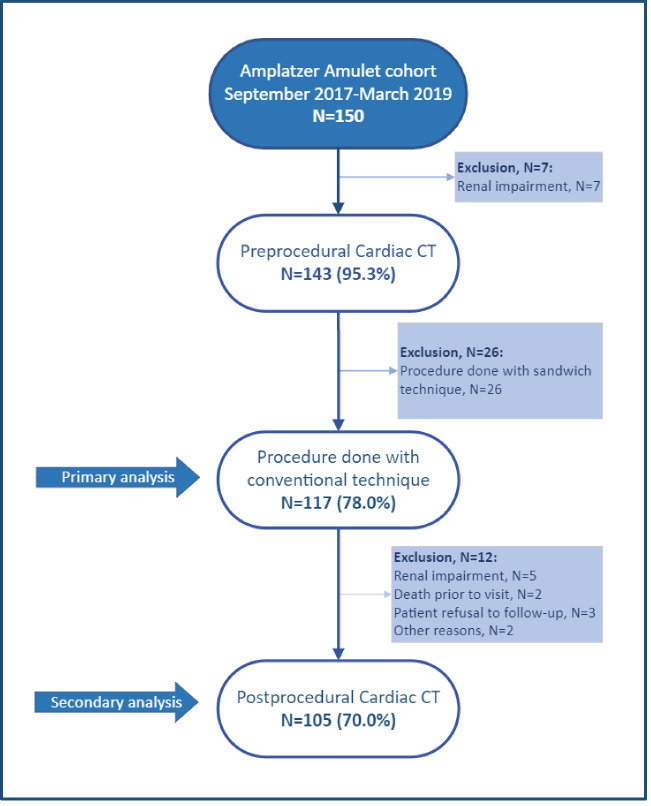
Flowchart for patient enrollment. All patients were included in the cohorts before acquiring the pre-procedural CT scans for LAAO. CT, computed tomography; LAAO, left atrial appendage occlusion. Graphics program, Microsoft Visio

This single-center retrospective observational study was based on 150 consecutive patients undergoing LAAO with the Amplatzer Amulet device at Aarhus University Hospital between September 2017 and March 2019. Patients were identified from a local prospective LAAO database. As per institutional protocol, preprocedural planning was done with cardiac CT, excluding patients with an estimated glomerular filtration rate < 30 mL/min (*n* = 7) **(**Fig. 1**)**. Patients were scheduled for a follow-up cardiac CT approximately 2 months after the procedure. A preprocedural cardiac CT was available for analysis in 143 patients, and 130 patients had follow-up cardiac CT.

The Amulet device is most often implanted in the neck of the LAA using a “conventional” implantation technique. In a subset of chicken-wing anatomies, the neck of the LAA is too short for a conventional implantation, and the lobe of the device is instead implanted in the chicken-wing itself using the so called “sandwich” technique. Sizing is fundamentally different for sandwich implantations, and patients implanted with this technique were excluded (*n* = 26) [17, 18].

### CT analysis

**Fig. 2:**
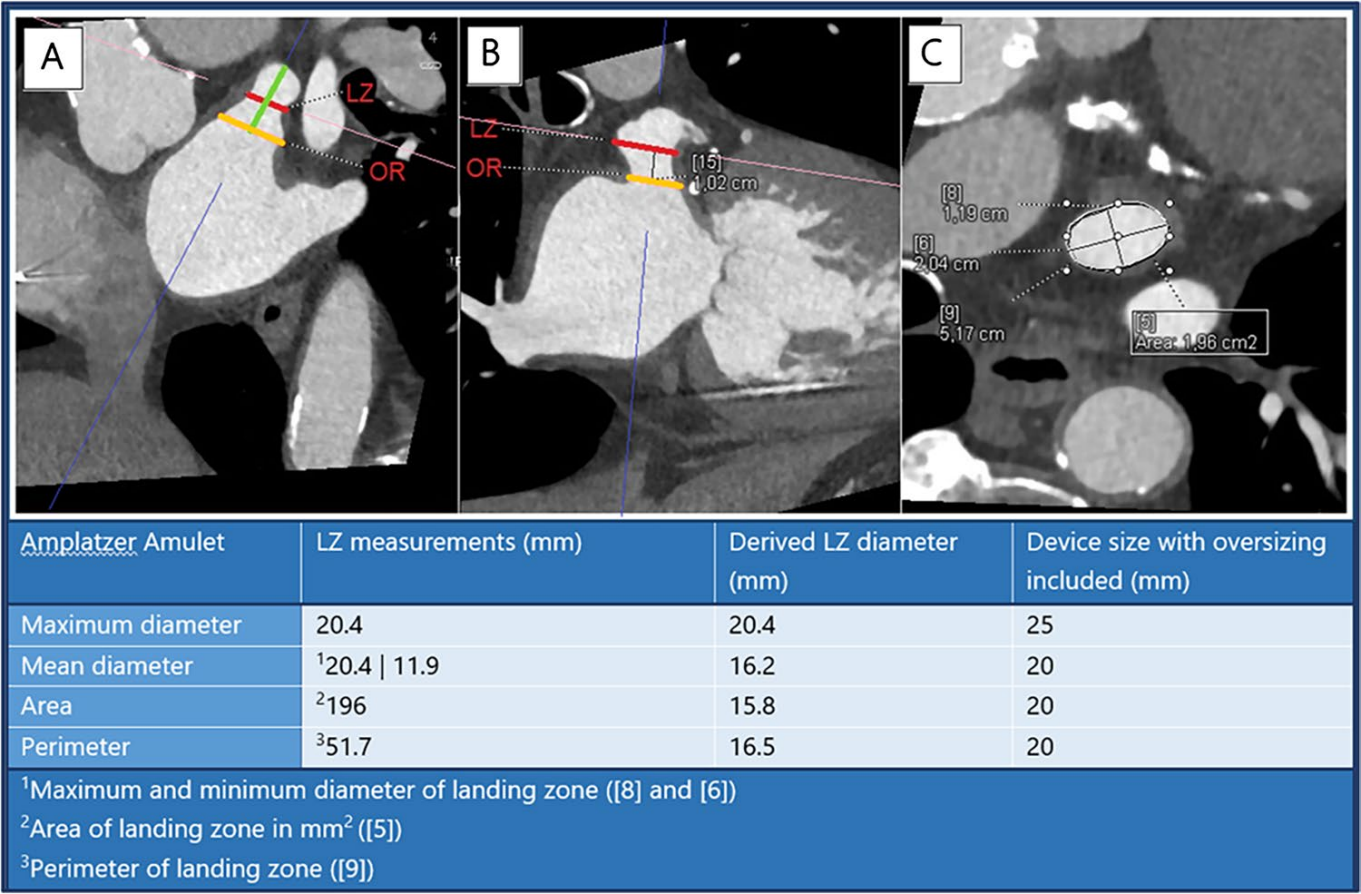
Multiplanar reconstruction of the left atrial appendage LAA orifice (yellow) and landing zone (red) measurements prior to Amulet implant (**A**-**C**). A two-chamber right-anterior oblique/caudal 30°/10° view of the LAA is generated (**B**), where the LAA orifice (yellow) is estimated from the circumflex artery to the pulmonary vein ridge. Landing zone is located 10–12 mm distal to the orifice, perpendicular to the LAA wall and presented in an en face projection (**C**). LAA depth (green) is estimated by a central, perpendicular line from the orifice to the backwall of the appendage. Landing zone measurements are converted from cm to mm. LZ, landing zone; LAA, left atrial appendage. Graphics program, Microsoft Word

Cardiac CT images were analyzed using syngo.via (Siemens Healthcare, Forchheim, Germany) by two investigators, both blinded to the implanted device sizes. A step-by-step approach to measure the LAA orifice and Amulet landing zone on cardiac CT has previously been described in detail [4]. In short, multiplanar reconstructed views were used to generate a two-chamber cardiac view resembling a 30°/10° right anterior oblique/caudal view. Here, the LAA and anatomical landmarks were identified, and crosshairs were placed to intersect the circumflex coronary artery and the left upper pulmonary vein ridge (LUPV ridge), defining the LAA orifice (Fig. 2).

The landing zone of the Amulet lobe was defined 10–12 mm distal to the orifice and perpendicular to the LAA wall. Depth of the appendage was assessed as a central, perpendicular line from the LAA orifice to the posterior wall of the appendage.

After segmenting images and identifying the predicted LAA landing zone, the minimum and maximum diameters were measured in an en face view, along with the area and perimeter of the landing zone. The mean diameter was calculated as the average of the measured maximum and minimum diameter. Area- and perimeter-derived diameter were calculated from the following formulas:

$$\begin{array}{c}area-derived\; diameter = 2 x \sqrt{\frac{area}{\pi }}\\ perimeter-derived\; diameter =\frac{circumference}{\pi }\end{array}$$

Device size was determined based on a 2–5 mm oversizing of the measured LAA landing zone diameter, as proposed in the manufacturer’s sizing algorithm [4] (Supplemental Fig 2). This approach was used across all four measures of the landing zone diameter.

The LAA eccentricity index was calculated according to the prior definitions used by Cho et al. [14]. Here, a large eccentricity index represents a more eccentric landing zone.

$$Eccentricity\; index=1-\frac{minimum \;diameter}{maximum\; diameter}$$

### Device sizing accuracy: prediction error

The primary outcome was the accuracy of each diameter to predict the actual implanted device size. Accuracy was estimated by the prediction error, calculated as the difference between the implanted device size and the predicted device size. Prediction error was calculated as both numerical prediction error (mean difference between the predicted and implanted device size) and absolute prediction error (mean value of absolute differences between the predicted and implanted sizes). A low prediction error was considered more accurate in terms of device-size accuracy. Based on the preprocedural CT measurements, the predicted device oversizing was calculated as the difference between predicted device size and estimated landing zone diameter. Similarly, the actual oversizing was estimated as the difference between the implanted device size and the estimated landing zone diameter.

### Peri-device leak

The association between prediction error and residual PDL was assessed on follow-up CT imaging. PDL was defined according to the presence of contrast patency and/or a visible channel connecting the left atrium and the LAA. Contrast patency was determined by a Hounsfield unit (HU) ratio between the LAA and left atrium of minimum 0.25 or Hounsfield units above 100 distal to the device. PDL was categorized according to a previously described algorithm [19]. In short, complete occlusion (grade 0) was defined as no visible PDL and no contrast patency in the distal LAA. PDL was categorized as grade 1 (contrast patency), grade 2 (visible PDL at disc level), or grade 3 (visible PDL at both disc and lobe). Only grade 3 PDL was considered clinically significant in the present study. Moreover, the mechanism of PDL was characterized according to a recently proposed classification [9, 19]. Accordingly, the underlying mechanism to all grade 3 leaks was assessed by three investigators and determined by consensus.

### Inter-rater variability

Inter-rater variability in predicting device landing zone and measuring LAA dimensions may affect device sizing and intraprocedural choice of device. Two investigators independently analyzed the same pre-procedural scans of 15 (10%) randomly selected patients. The maximum, mean, area-, and perimeter-derived diameters were measured and compared between each investigator by visual inspection of Bland–Altman plots [20, 21].

### Statistics

Distribution of data was evaluated by histograms and Q-Q plots. Continuous variables were expressed as mean (± SD) and were compared using student’s *t* test or paired *t* test, as appropriate. Categorical variables were presented as counts (percentages) and compared by using the Pearson’s chi-square. Receiver operating characteristic (ROC) analysis was used to estimate an optimal landing zone eccentricity cut-off. Bland–Altman plots were used to investigate inter-reader variability. All statistical analysis was conducted using STATA version 17 (Statacorp, College Station, Texas).

## Results

A total of 143 (95.3%) patients had pre-procedural CT available (Fig. 1**)**. A conventional implantation technique was used in 117/150 (78%), and this cohort of patients was included in the analysis of device-sizing accuracy. The sandwich technique was used in 26/150 (17.3%), and those patients were excluded from the cohort. For the analysis on PDL, a postprocedural follow-up CT was available in 105/117 (89.70%) patients with a conventional implant.

Baseline characteristics are presented in Table 1. Chicken-wing (39.3%) and windsock (47.9%) LAA morphologies were frequent, and the landing zone was predominantly eccentric (59.1%) (Table 2). Procedures were guided by intracardiac echocardiography in 105/117 (89.7%). The mean implanted device size was 25.0 mm (± 3.9 mm), and in 110/117 (94.0%), the first selected device was implanted.

**Table 1:** Baseline characteristics

Baseline characteristics	
	*N* $=$ 117 (78.0%)
Age at LAAO, mean (**± **SD)	73.1 ± 9.9
Male	79 (67.5%)
BMI, mean (**± **SD)	27.1 ± 5.0
CHA_2_DS_2_-VASc score, mean (**± **SD)	3.95 ± 1.52
HAS-BLED score, mean (**± **SD)	2.66 ± 0.87
Indication for LAAO^1^	
HAS-BLED score ≥ 3	59 (50.4%)
History of intracranial bleeding	21 (17.9%)
History of gastrointestinal bleeding	27 (23.1%)
History of urinary tract bleeding	10 (8.5%)
History of other spontaneous bleeding	34 (29.1%)
Cerebral amyloid angiopathy	4 (3.4%)
Stroke despite OAC/NOAC	15 (14.6%)
Preference/compliance^2^	9 (7.7%)
Other indications^3^	34 (29.1%)

Unless stated otherwise, data are shown as *n* (%). *OAC* oral anticoagulant therapy, *NOAC* novel oral anticoagulant, *LAAO* left atrial appendage occlusion

^1^Patients may have more than one indication for LAAO

^2^Patient preferring LAAO over anticoagulation or lack of compliance to OAC/NOAC

^3^Other indications: anemia, renal insufficiency, hemophilia A, and inclusion in other studies

**Table 2:** Preprocedural and procedural characteristics

Preprocedural characteristics	*N* $=$ 117 (78.0%)
LAA morphology	
Chicken-wing morphology	46 (39.3%)
Windsock morphology	56 (47.9%)
Cactus morphology	10 (8.5%)
Cauliflower morphology	5 (4.3%)
LAA landing zone shape	
Eccentric	65 (59.1%)
Round	32 (29.1%)
Triangular	5 (4.5%)
Foot-like	5 (4.5%)
Waterdrop	3 (2.7%)
LAA depth, mean (± SD) (mm)	
	22.0 ± 5.0
Implanted device size, mean (± SD) (mm)	
	25.0 ± 3.9
Procedural characteristics	
Contrast use, mean (± SD) mL	56.6 ± 23.4
Procedural time, mean (± SD) (min)	37.5 ± 16.1
Procedural guidance	
ICE	105 (89.7%)
TEE	12 (10.3%)
Number of devices used	
1	111 (94.9%)
2	5 (4.3%)
3	1 (0.9%)
Number of attempts	
1	110 (94.0%)
2	6 (5.1%)
3	1 (0.9%)

Unless stated otherwise, data are shown as *n* (%). Amulet implantations with sandwich technique are excluded. *LAAO* left atrial appendage occlusion, *ICE* intracardiac echocardiography, *TEE* transesophageal echocardiography

### Prediction error

The numerical prediction error was greatest when using maximum diameter at − 2.55 mm (± 2.57) (Table 3). In comparison, calculated diameters (mean, area-, and perimeter-derived diameters) were more accurate in predicting the actual implanted device size with numerical prediction errors of 0.08 mm (± 2.77), 0.30 mm (± 2.40), − 0.39 mm (± 2.43), respectively. Likewise, the absolute prediction error of the maximum diameter was significantly larger compared to the calculated parameters that all displayed similar absolute prediction errors. On average, the maximum diameter was significantly larger than the calculated parameters, with 23.0 mm (± 4.9) compared to 20.4 mm (± 4.5), 20.3 mm (± 4.4), and 21.1 mm (± 4.4) (Table 3). Predicted oversizing was similar across maximum, mean, and area-derived diameter, although smaller for the perimeter-derived diameter. Actual oversizing appeared smallest for the maximum diameter.

**Table 3:** Measured diameters and predicted device sizing

*N* $=$ 117	Mean (± SD) (mm)	Numerical prediction error (± SD) (mm)	Absolute prediction error (± SD) (mm)	Predicted oversizing (± SD) (mm)	Actual oversizing (± SD) (mm)
Maximum diameter	23.0 ± 4.9	− 2.55 ± 2.57	2.74 ± 2.36	4.54 ± 1.46	1.97 ± 3.06
Mean diameter	20.4 ± 4.5***	0.08 ± 2.77***	1.83 ± 2.07**	4.52 ± 0.89	4.62 ± 2.67***
Area-derived diameter	20.3 ± 4.4***	0.30 ± 2.40***	1.65 ± 1.77***	4.36 ± 0.85	4.67 ± 2.33***
Perimeter-derived diameter	21.1 ± 4.4***	− 0.39 ± 2.43***	1.75 ± 1.72***	4.22 ± 0.90*	3.84 ± 2.41***

Data are presented as millimeters and as mean (± SD). Mean, area-derived, and perimeter-derived diameter was compared with the maximum diameter using unpaired *t* test. *p-value < 0.05, **p-value < 0.005, ***p-value < 0.0005

**Fig. 3:**
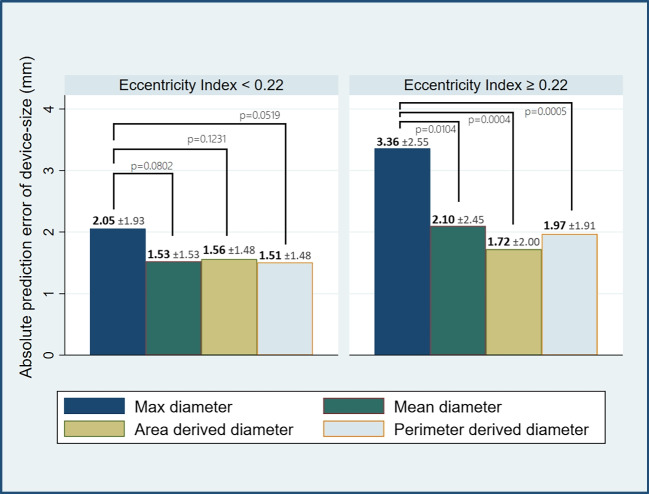
Absolute prediction error for the four measured diameters in landing zones of eccentricity index: < 0.22 and ≥ 0.22. Maximum diameter has been statistically compared to mean, area-, and perimeter-derived diameter using *t* tests. Data are shown as mean (± SD). Graphics program, Stata 17

The optimal cut-off points for eccentricity index could not be readily identified by ROC curves (Supplemental Fig 3). Thus, the patient cohort was split into two groups at the 50% percentile of eccentricity index (< 0.22 and ≥ 0.22). Calculated diameters consistently displayed the lowest prediction error regardless of eccentricity status compared to maximum diameters (Fig. 3). At eccentricity index ≥ 0.22, the area-derived diameter achieved the lowest absolute prediction error at 1.72 mm. When using maximum diameter, the absolute prediction error increased significantly in landing zones of eccentricity index ≥ 0.22 vs < 0.22, respectively (Table 4). Similar differences were not detected in derived diameters. In low-eccentricity landing zones (EI < 0.22), maximum diameter tended to perform worse than the calculated diameters. Based on scatter plot analysis, the overall prediction error did not seem to increase with increasing eccentricity of the landing zone (Supplemental Fig 4).

**Table 4:** Absolute prediction error in relation to eccentricity index

Absolute prediction error (*N* $=$ 117)	Eccentricity index < 0.22 (± SD) (mm) (*N* $=$ 55)	Eccentricity index ≥ 0.22 (± SD) (mm) (*N* $=$ 62)
Maximum diameter	2.05 ± 1.94	3.36 ± 2.55**
Mean diameter	1.53 ± 1.53	2.10 ± 2.45
Area-derived diameter	1.56 ± 1.48	1.72 ± 2.00
Perimeter-derived diameter	1.51 ± 1.48	1.97 ± 1.91

Data are presented as millimeters and as mean (± SD). Absolute prediction error for each four measured diameters compared in eccentricity index of < 0.22 and ≥ 0.22 using simple *t* tests. *p-value < 0.05, **p-value < 0.005, ***p-value < 0.0005

### Peri-device leak

At 2-month follow-up, contrast patency was a common finding (*n* = 54, 51.4%), and approximately half the patients had a PDL at the device disc (*n* = 51, 48.6%). Grade 3 PDL was seen in nine (8.7%) patients (Table 5).

**Table 5:** PDL grade at 8-week follow-up cardiac CT

	*N* $=$ 105 (70.0%)
PDL	
Complete occlusion	36 (34.3%)
Grade 1 leak	16 (15.2%)
Grade 2 leak	44 (41.9%)
Grade 3 leak	9 (8.5%)
Grade leak criteria	
Contrast patency^1^	54 (51.4%)
PDL at device disc	51 (48.6%)
PDL at device lobe	9 (8.6%)

PDL was categorized into complete occlusion and grade 1–3 leak. *PDL* peri-device leakage, *LAA* left atrial appendage, *LA* left atrium

^1^Contrast patency defined as contrast ratio between LAA: LA as > 0.25 or contrast found distal to the device > 100 Hounsfield units

Analysis of the underlying leak mechanism of grade 3 leaks showed device malalignment (*n* = 5), device undersizing (*n* = 2), uncovered lobe (*n* = 1), and one inconclusive.

The LAA landing zone diameters appeared larger in patients with grade 3 PDL, reaching statistical significance across all parameters (Table 6). The numerical prediction error was significantly larger for area and perimeter-derived diameters, while not significantly different for maximum and mean diameters. Landing zone eccentricity was not significant in higher degrees of PDL.

**Table 6:** Comparison of PDL grade 0 vs PDL grade 1–2 vs PDL grade 3 in conventional technique

	PDL grade 0	PDL grade 1–2	PDL grade 3	*p*-value
$n=$ 105	*N* $=$ 36 (34.3%)	*N* $=$ 60 (57.1%)	*N* $=$ 9 (8.6%)	
Mean (mm) (± SD)				
LZ max diameter	21.0 ± 4.8	23.8 ± 4.5	26.4 ± 6.0	0.003
LZ mean diameter	18.3 ± 4.6	21.3 ± 4.0	23.0 ± 5.6	0.002
LZ area-derived diameter	18.1 ± 4.4	21.4 ± 3.9	22.8 ± 5.4	0.001
LZ perimeter-derived diameter	19.1 ± 4.4	22.1 ± 4.0	23.7 ± 5.7	0.001
Numerical prediction error (mm) (± SD)				
Max diameter	− 2.49 ± 2.96	− 2.20 ± 2.35	− 3.67 ± 2.50	0.278
Mean diameter	0.63 ± 2.53	0.15 ± 2.85	− 1.78 ± 3.23	0.073
Area-derived diameter	1.06 ± 2.20	0.17 ± 2.46	− 1.11 ± 2.42	0.036
Perimeter-derived diameter	0.23 ± 2.34	− 0.44 ± 2.34	− 2.42 ± 3.18	0.036
Average eccentricity index (± SD)				
	0.26 ± 0.13	0.20 ± 0.14	0.26 ± 0.06	0.088

Landing zone size and numerical prediction error was assessed in PDL grade 0 vs PDL grade 1–2 vs PDL grade 3. Average eccentricity index was estimated. Data are presented as mean (± SD) and *n* (%). *PDL* peri-device leakage, *LZ* landing zone

### Inter-rater variability

**Fig. 4:**
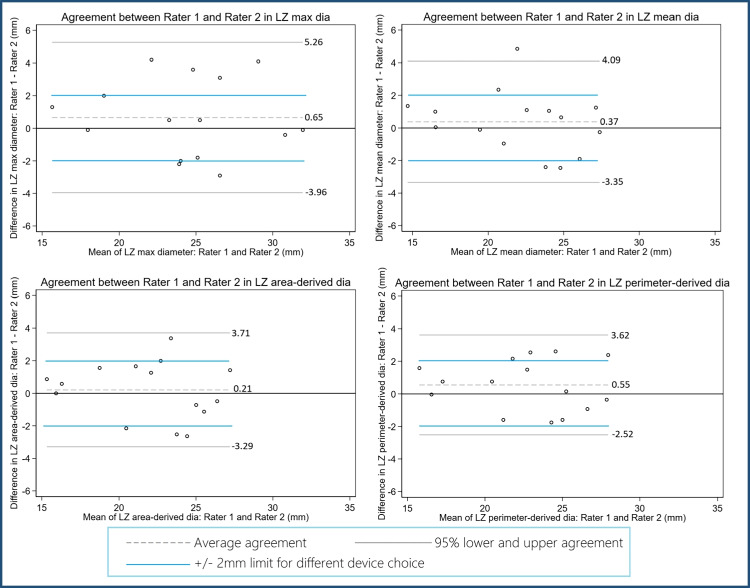
Interobserver variability in Bland–Altman plots. Two investigators analyzed preprocedural cardiac CT images of 15 Amulet implantations. CT, computed tomography; LZ, landing zone. Graphics program: Stata 17 and Microsoft Paint

The inter-reader analyses revealed a moderate agreement with a minor systematic bias of less than 0.6 mm between investigators across all four parameters (Fig. 4). Systematic bias tended to be lower in mean, area-, and perimeter-derived diameter. The limits of agreement were moderately wide, up to 5.26 mm for LZ max diameter, and with the narrowest limit of agreement using perimeter-derived diameter. Systematic bias tended to be lower in mean, area-, and perimeter-derived diameter. These limits of agreement in all four parameters exceeded 2 mm, potentially prompting a difference of one or more device sizes. One observation was detected outside of mean ± 2SD.

## Discussion

In this retrospective single-center cohort study, we investigated four different measures of the LAA landing zone diameter and their ability to predict the implanted Amulet device size. Our results suggest that the maximum diameter may be less accurate than calculated diameters (mean, area-derived, and perimeter-derived diameters), especially in eccentric landing zones. Compared to the maximum diameter, all calculated diameters appeared equally good for device-size prediction, and their accuracy seemed less reliant on landing zone eccentricity. Predicted oversizing was equal across all four parameters; however, actual oversizing appeared smallest for the maximum diameter compared to the others. This indicates that maximum diameter may tend to oversize the device, when using the manufacturer’s sizing algorithm of 2–5 mm oversizing.

### Current knowledge on device-sizing

Previously, Cho et al. [14] demonstrated how minimum diameter as a stand-alone measure could be dismissed when the eccentricity of the landing zone increased. In theory, device sizing solely based on minimum diameter would lead to severe under-sizing, potentially increasing the risk of PDL in eccentric LAA landing zones. Cho et al. concluded that the perimeter-derived diameter was significantly more accurate in device-size prediction regardless of eccentricity index, as compared with maximum, mean, and area-derived diameters in Amplatzer Cardiac Plug and Amulet devices. Wang et al. [6] found the perimeter-derived diameter to be more reproducible when sizing the LAA orifice compared to maximum and minimum diameter. Our results are in agreement with previous findings, indicating maximum diameter alone may be an insufficient measure, when determining device size. Consensus in Amulet sizing has been to oversize the device by 2–5 mm based on landing zone diameter (Supplemental Fig 2). A position paper on device-sizing with the Amulet device, however, suggested mean diameter might be a better alternative if 3D imaging is available, while stressing that the selected device should not be smaller than the maximum diameter [15]. The predicted oversizing was similar across all four parameters in our study, yet the actual oversizing was smaller for the maximum diameter. This may indicate that choosing a device with oversizing at the upper level of the suggested 2–5 mm range relative to the maximum diameter alone may significantly oversize the device. Incorporating both the maximum and minimum diameter into the mean diameter, or alternatively calculating perimeter- or area-derived diameters, may provide a better approximation of an eccentric landing zone, which is also supported by recent studies [15, 16].

### Deciding factors in device-sizing

It should be acknowledged that device-sizing in LAAO is complex with the anatomical heterogeneity of the LAA adding to the challenge of planning the procedure. Even small changes in the angulation or depth of the predicted landing zone may impact the measures. Similarly, transseptal puncture, sheath selection, and orientation of the LAA may affect the intraprocedural ability to position the device in the exact same position as predicted during preprocedural CT. Moreover, equally optimal implantation results may likely be achievable with two neighboring device sizes. Consequently, the feasibility of standardizing device sizing into intervals of diameter measurements is questionable. Many factors are at play during the overall process from preprocedural planning to the final implantation, including observer variance during preprocedural planning and operator preference.

In the interobserver analysis, in line with prior studies, we documented a minor systematic bias across all four parameters, while the limits of agreement indicated a moderately large discrepancy between investigators. Of note, the limits of agreement appeared widest for sizing based on the maximum diameter. One way of reducing human error in preprocedural planning may be the use of advanced, dedicated software for planning the intervention, and AI-assisted computational modeling may prove beneficial to optimize the procedural efficiency and result [13]. There is increasing evidence highlighting the importance of position, depth, and sealing of the device to reduce the risk of device-related thrombosis and PDL, which, in turn, may reduce the residual risk of thromboembolism after LAAO [9, 10, 22].

## Limitations

In the current study, predicted device size was estimated from measured maximum diameter intervals based on the manufacturers’ sizing algorithm. This guideline for the Amulet is based on non-overlapping intervals. Consequently, few millimeters of miss-sizing can impact the recommended device size. In this situation, other factors such as patient history, risk factors, and LAA anatomy may be granted greater significance in the decision between the smaller or larger device size. In clinical practice and according to manufacturer’s recommendation (Supplemental Fig 2), operators often oversize the Amulet to ensure stability. During CT-based preplanning, few degrees of rotation can create a vastly different view and measurement of the landing zone. Thus, device sizing is reliant on experience in both planning and implantation.

The sizing algorithm for Amulet used in this study is based on maximum diameter measured by TEE. As previously shown, there is a potential large discrepancy between landing zone measuring with CT and TEE [23]. Lastly, a retrospective study has inherent limitations and does not allow conclusions regarding causality. This study is based on a single high-volume center with experience in cardiac CT interpretation related to LAAO. Consequently, our results might not translate to other health centers and device types.

Further prospective studies on this subject are warranted.

## Conclusion

This study shows a large device size prediction error when solely relying on maximum LAA landing zone diameter. Alternative measures like mean, area-, or perimeter-derived diameter may prove better at predicting the optimal LAAO device size with the Amplatzer Amulet. Prospective, randomized trials are needed to clarify the optimal sizing algorithm.

### Supplementary Information

Below is the link to the electronic supplementary material.

Supplementary file1 (DOCX 642 KB)

## Data Availability

All image-related data were stored using REDCap (Vanderbilt University, Nashville, Tennessee), while clinical information from patient files was stored in a web-based electronic case report form system (TrialPartner). Statistical analysis was performed using STATA (STATA IC, version 17, StataCorp, College Station, Texas). Study data are available from the corresponding author upon reasonable request. However, due to data sensitivity, a full dataset will not be publicly available.

## Abbreviations

AF: Atrial fibrillation
CT: Computed tomography
LAA: Left atrial appendage
LAAO: Left atrial appendage occlusion
PDL: Peri-device leak
LUPV: Left upper pulmonary vein
