# Supplementary material for: Computed tomography–based device-sizing in Amplatzer Amulet left atrial appendage occlusion
Source: J Interv Card Electrophysiol. 2023 Oct 26;67(4):785–95. doi: 10.1007/s10840-023-01665-y (PMC11166793; doi:10.1007/s10840-023-01665-y)
Supplement: Supplementary file 1 — Supplementary file1 (DOCX 642 KB) [file 10840_2023_1665_MOESM1_ESM.docx]

**Article** **Title**: Computed Tomography Based Device-sizing in Amplatzer Amulet Left Atrial Appendage Occlusion

**Journal** **Name**: Journal of Interventional Cardiac Electrophysiology

**Author** **names**: Jonatan Gerard Nirmalan, BSc^a^; Anders Kramer, MD^a^; Kasper Korsholm, MD, PhD^a^; Jesper Møller Jensen, MD, PhD^a^; Jens Erik Nielsen-Kudsk, MD, DMSc^a^

**Affiliation**: ^a^Department of Cardiology, Aarhus University Hospital, Aarhus, Denmark

**Address** **for** **correspondence**:

Jens Erik Nielsen-Kudsk, Prof., MD, DMSc

E-mail: [je.nielsen.kudsk@gmail.com](mailto:je.nielsen.kudsk@gmail.com)

**Content:** Supplementary figures and tables

# Supplemental

**Fig. 1**


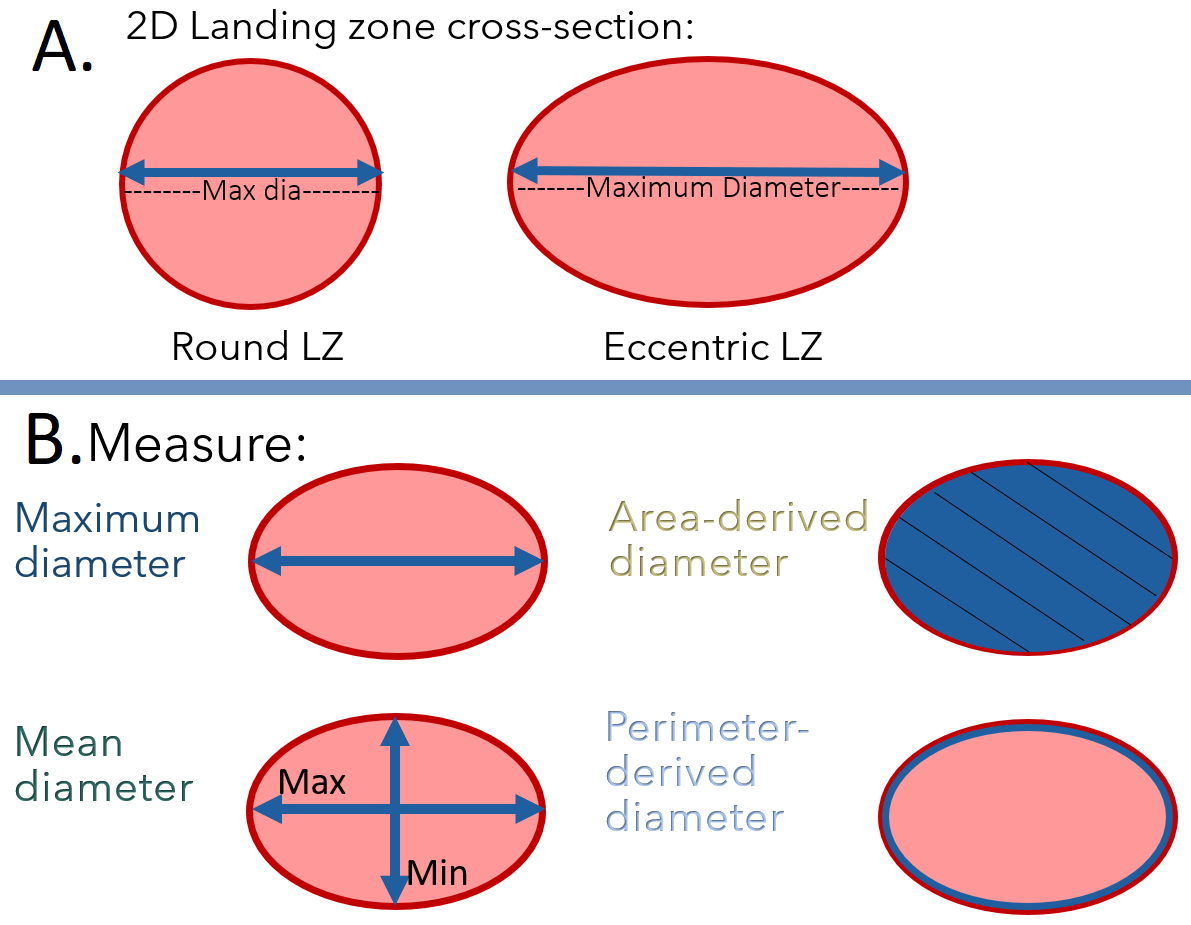


A: Maximum diameter as measured in a circular and a highly eccentric landing zone B: Maximum diameter and calculated diameter dimensions.

Graphics program: MS Powerpoint

**Fig. 2**


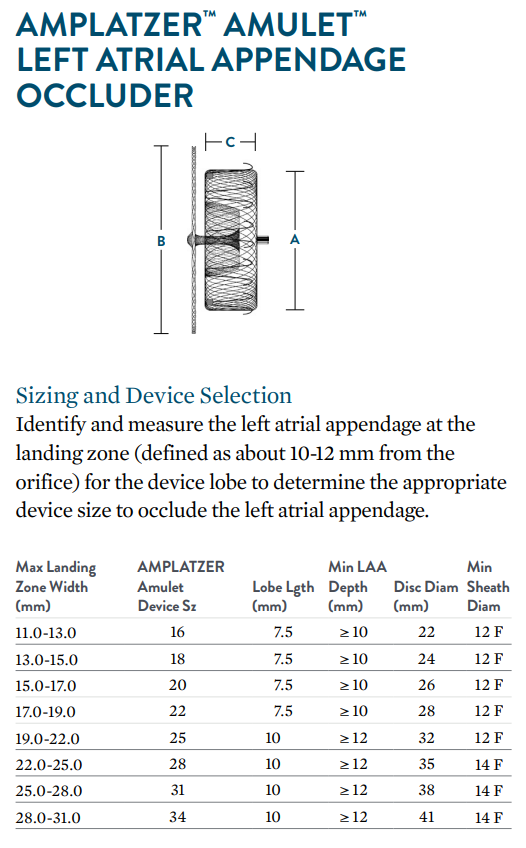


AMPLATZER™ PRODUCT PORTFOLIO. Device-sizing chart. *LAA* Left Atrial Appendage
<https://www.cardion.cz/fil/703/Amplatzer>

**Fig. 3**


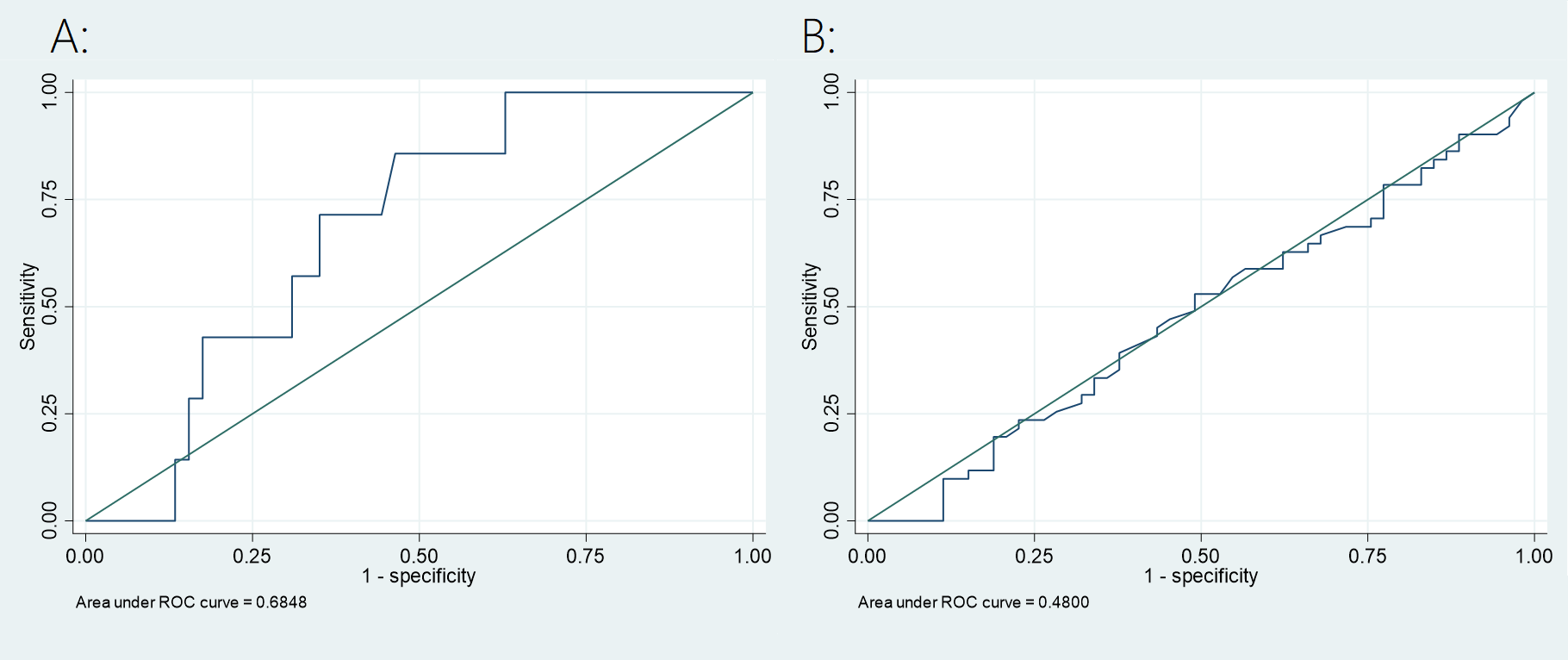


Receiver Operating Characteristic (ROC) curves for peridevice leak (PDL) grade versus eccentricity index. ROC analysis was done for A) PDL grade ≤ 2 and B) PDL grade ≤ 1

Graphics program: Stata 17

**Fig. 4**


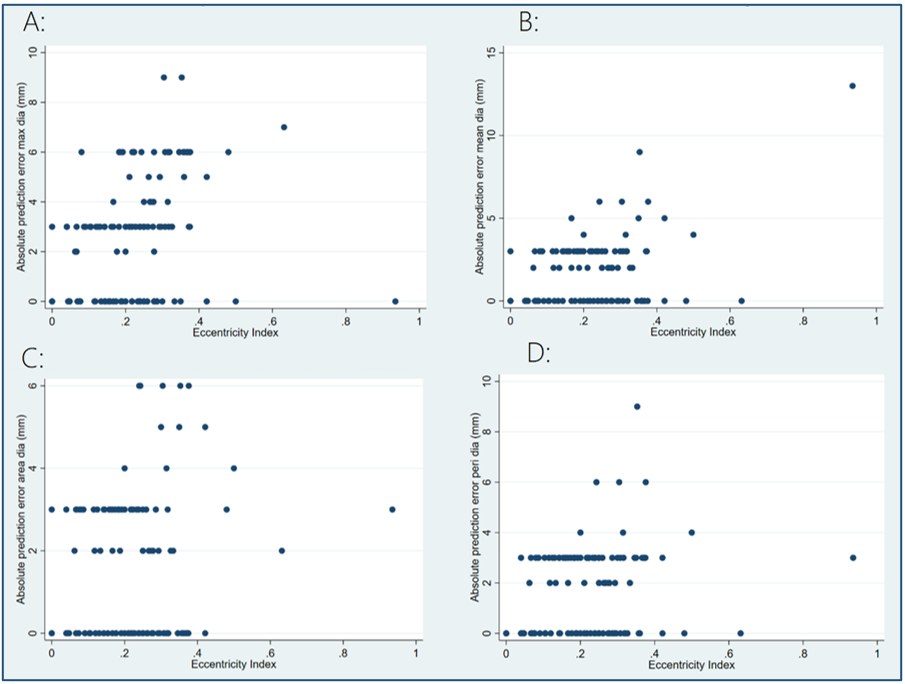
Scatter-plot analysis. Correlation between absolute prediction error and eccentricity index for A: maximum diameter, B: mean diameter, C: area-derived diameter, D: perimeter-derived diameter.

Graphics program: Stata 17
